# Self-Medication with Antibiotics during COVID-19 in the Eastern Mediterranean Region Countries: A Review

**DOI:** 10.3390/antibiotics11060733

**Published:** 2022-05-30

**Authors:** Feras Jirjees, Munazza Ahmed, Somayeh Sayyar, Monireh Amini, Hala Al-Obaidi, Mamoon A. Aldeyab

**Affiliations:** 1 Department of Pharmacy Practice and Pharmacotherapeutics, College of Pharmacy, University of Sharjah, Sharjah P.O. Box 27272, United Arab Emirates; munazza.ahmed@sharjah.ac.ae (M.A.); u17102946@sharjah.ac.ae (S.S.); u17100469@sharjah.ac.ae (M.A.); 2 College of Pharmacy and Health Sciences, Ajman University, Ajman P.O. Box 346, United Arab Emirates; h.alobaidi@ajman.ac.ae; 3 Department of Pharmacy, School of Applied Sciences, University of Huddersfield, Huddersfield HD1 3DH, UK; m.aldeyab@hud.ac.uk

**Keywords:** self-treatment, antibiotic, Eastern Mediterranean Region, COVID-19

## Abstract

Self-treatment with medicines including treatment with antibiotics is a growing global concern, as it can cause public health problems, such as antibiotic resistance and drug toxicity. Therefore, the significance of the self-medication impact of COVID-19 in any region can have an influence on the prevalence of such problems. The review aimed to investigate the self-treatment with antibiotics among the general population in Eastern Mediterranean region countries during COVID-19 pandemic. A comprehensive review of literature in four databases was conducted for the pandemic period from January 2020 to the end of March 2022. Nine studies related to self-treatment with antibiotics were found. The studies were homogeneous in terms of assessing the antibiotic self-treatment usage during the COVID-19 pandemic among the general population and among community pharmacies. The prevalence of self-treatment with antibiotics ranged from 20.8% to 45.8% between the studies. The main reasons for that were cost-saving, fear of COVID-19 infection, quarantine, and ease of accessibility without time limits. Antibiotic self-treatment has been high during the COVID-19 pandemic; however, it was less reported during the study period than before the time of the pandemic. There is a need for more restrictions on dispensing antibiotics from community pharmacies. In addition, there is a need to raise awareness among the population regarding self-treatment with antibiotics.

## 1. Introduction

Globally, self-treatment with medicine is a growing phenomenon in all societies with the prevalence reaching over 90% in some societies, which is highly dependent on the target population and country, diseases, and medications [1,2]. According to the World Health Organization (WHO) guideline, self-treatment with medicine is an act of irregular and persistent use of prescribed medicine for recurrent or chronic symptoms and self-diagnosed diseases. Additionally, attaining medicines without prescription, reclaiming outdated prescriptions to purchase medicines, sharing medicines between one’s relatives, and intake of medicine surpluses stored at home have all been attributed to self-medication [3]. Although most pharmaceutical regulations around the world prohibit many medicines from being dispensed without a prescription, a proportion of global health practices lack control over self-treatment with prescription medicines, including antibiotics [3]. Self-medication with antibiotics (SMA) has been documented to cause complications, such as adverse effects, therapeutic failure, and, most significantly, antibiotic resistance [4,5,6]. Several published studies reported on the improper antibiotic dispensing practices worldwide, an issue that is prevalent in several Eastern Mediterranean Region (EMR) countries [7,8,9]. A systematic review, that assessed antibiotics usage outside official healthcare facilities in low middle-income countries within the Middle East region, showed that the prevalence of SMA was as high as 82%, with 20% to 50% being improper usage [7].

The emergence of COVID-19 in the first months of 2020 has resulted in a substantial increase in the injudicious use of antibiotics and was associated with a rise in SMA in many developing countries in an attempt to protect themselves from the virus [9]. Although antibiotics do not treat or prevent viral infections, including COVID-19, studies showed that along with the surge in infectious cases, a parallel rise in antibiotic usage was reported [10,11,12,13,14]. In a study among those taking antibiotics, around 80% were not infected with COVID-19 and were inappropriately taking antibiotics in the hopes of preventing the infection. Moreover, although only a low ratio (15%) of those severely affected with COVID-19 developed bacterial co-infection, causing the need to initiate antibiotics, 75% of them ended up receiving antibiotics [12]. The absence of approved treatment for COVID-19 infected patients and the role social media has in creating panic among people have been contributing factors in dramatically increasing the prevalence of SMA [15].

Most global and national guidelines for managing patients infected with COVID-19 with antibiotics have not been addressed, either for self-treating or for hospitalized patients, unless there is a secondary bacterial co-infection [14,15]. Based on the WHO guidelines, antibiotics should be given in secondary bacterial complications in moderate to severe COVID-19 cases [15]. However, a low ratio of patients infected with COVID-19 might suffer from bacterial co-infection as well [16,17]. Hence, the definition of antibiotic misuse in the COVID-19 pandemic emerged as the prescription and dispensing of antibiotics without indication.

### 1.1. Self-Medication with Antibiotics before the Era of COVID-19 in EMR Countries

Several published systematic reviews conducted on SMA before COVID-19 in EMR countries showed that the use of antibiotics without prescriptions or self-treatment was a common practice [4,6]. The high prevalence of SMA (between 19% and 82%) was reported in many countries in this region, including Yemen, Oman, Saudi Arabia, United Arab Emirates (UAE), Syria, Iran, Jordan, Lebanon, and Turkey [4,18]. Furthermore, common antibiotic classes associated with SMA reportedly were penicillin, cephalosporin, fluoroquinolones, metronidazole, and tetracycline for various indications [8], with the most common indication for self-medication being upper-respiratory-tract infection [18].

Multiple factors associated with SMA include age, sex, income level, and educational status [4]. Higher self-medication rates between individuals with low education levels and low- or middle-income have been reported. In addition, the lack of proper information about antibiotics’ role in treatment led to irrational use of antibiotics among people [4]. This led to inappropriate practices of shorter duration of treatment, wrong doses, and drug intake for the wrong indication, for instance, using antibiotics to treat viral infections [19].

### 1.2. Regulations of Prescribed Antibiotic in the EMR Countries

Several regulations and policies related to prescribing antibiotics in the EMR countries highlight the importance of abiding by the health regulations and prohibit pharmacists to dispense antibiotics without a prescription specifically issued by a doctor licensed to practice within the country [20,21,22].

The study aimed to investigate the self-use of antibiotics among people of EMR countries and healthcare providers during COVID-19 by reviewing the literature published on this topic.

## 2. Method

The search criteria included studies published during the COVID-19 pandemic period in the last two years, i.e., from 1 January 2020 to 31 March 2022 in four big databases: PubMed, Scopus, Medline, and Google Scholar. Four groups of keyword terms have been used: the first group included “Middle East”, “Eastern Mediterranean”, Afghanistan, Bahrain, Djibouti, Egypt, Jordan, Iran, Iraq, Kuwait, Oman, Qatar, Saudi, Lebanon, Libya, Morocco, Palestinian, Somalia, Sudan, Syria, Tunisia, “United Arab Emirates”, UAE, and Yemen. These countries were listed because they comprise the EMR based on WHO EMRO and consist of 21-member states [23]. Additional terms searched included Algeria and Mauritania. The second term search group included was: COVID, COVID-19, and CORONA. While the third terms search group included: pharmacist, doctor, physician, nurse, healthcare, “health care”, pharmacy, and outpatient. The fourth terms search group utilized in the search strategy was: antibiotic, antibacterial, anti-bacterial, antiviral, “self medication”, self-medication, self-treatment, “self treatment”, SMA, misuse, and abuse. Additionally, the most common antibiotics dispensed via community pharmacies were used in the search: azithromycin, amoxicillin, and erythromycin.

Exclusion criteria involved: studies covering data prior to 2020, studies not including self-treatment with antibiotics, and studies conducted in countries other than those in the EMR.

## 3. Results

**Figure 1:**
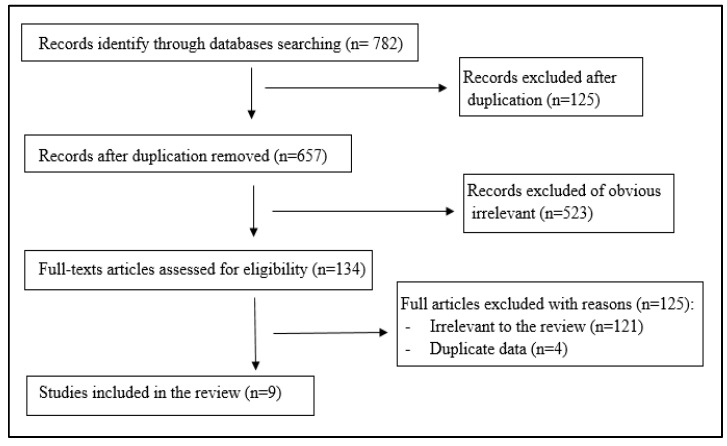
Flowchart of studies included in the review.

The electronic search of the four databases identified 782 records; only nine studies related to the SMA topic or inappropriate antibiotics prescribed during COVID-19 were included. These studies were conducted in six countries (belong to the EMR): two studies each in Jordan, Iran, and Pakistan; and one study each in Egypt, Iraq, and Saudi Arabia. The study design of all was a cross-sectional study. Figure 1 presents the flow diagram of the literature search process.

The studies included in this review were homogeneous in terms of assessing the antibiotic proper usage during the COVID-19 pandemic among general population and among community pharmacies.

### 3.1. Self-Medication with Antibiotics among People during COVID-19 in the EMR Countries

Table 1 shows the characteristics and the main findings of included studies related to SMA during the COVID-19 pandemic among general population in some EMR countries. The two published studies in Pakistan found that 53% of the participants took the drugs from different sources, such as family, friends, and/or directly from the community pharmacies. A total of 10.7% of the participants had the habit of taking self-medication and 16.3% of respondents did self-medicate with antibiotics for fear of contracting the virus. Azithromycin accounted for 21.5% of the self-medicated drugs [24]. In the second study, about a third of the participants had self-treatment with antibiotics; azithromycin was the most self-medicated antibiotic among more than a quarter of participants, and doxycycline was used by about 4% of them [25].

In Iran, there were also two studies. The rate of arbitrary drug use in the first study was 56.4%, whereas the percentage of SMA in this population was 27.1 [28]. In the second study, 20.8% of the participants took antibiotics during the pandemic without physicians’ prescription, and there was an overall decrease in SMA during the COVID-19 pandemic, with 38.1% reported prior COVID-19 period [29].

In a study conducted in Iraq, 45.8% of the participants reported SMA without a physician’s prescription. The main reasons were flu/common cold and sore throat [26]. The ratio of SMA decreased in Jordan as reported by a study (*n* = 1179) with around 30% of participants self-medicated on antibiotics during COVID-19; the main causative factors of such being to treat symptoms such as fever, muscle pain, and sore throat [27].

### 3.2. Dispensing Antibiotics during COVID-19 in the Community Pharmacies

Table 2 shows three studies that assessed the usage of antibiotics during the COVID-19 pandemic in community pharmacies. The first published study conducted in Egypt reported that 18% of the dispensed antibiotics were given without prescriptions and comprised upon both pharmacists’ recommendations and patients’ requests [30]. Azithromycin was the most common antibiotic (48%) dispensed in the community pharmacies and was given to around 40% of presumptive patients showing only mild or moderate symptoms for 5–10 days. In addition, all prescriptions were issued from private clinics and antibiotic combinations were given to 74% of home-isolated patients for a maximum of two weeks [30]. The second study conducted in Saudi Arabia and stated that 15.8% of pharmacists were convinced to sell non-prescribed antibiotics to the demands of the customers; however, there are restrictions on dispensing antibiotics without prescriptions that have been developed in the recent years [31]. In the third study, in Jordan, the dispensing of azithromycin without prescriptions was significantly higher during COVID-19, 127% higher than before the pandemic [32].

## 4. Discussion

This review reported the frequencies of self-medication with antibiotics from selected studies in the EMR countries (between 20% to 45%) and showed some patterns of SMA could be deduced. Self-medication to prevent or treat COVID-19 has become quite common among the public [33,34,35]. The rapid transmission and severity of COVID-19 disease sparked fear and stress among people worldwide due to the lack of definitive treatment and the disease’s elusive nature. In addition, the situation has been exaggerated by misinformation via various social media platforms [12].

There is a lack of information about antimicrobial consumption among most EMR countries. There is only one study about general antimicrobial consumption in Jordan to assess the effect of the COVID-19 pandemic on antimicrobial consumption based on reports obtained from the Jordan Drug and Food Administration (JDFA) among Jordanians (more than 10.5 million people). Surprisingly, the total antimicrobial usage has decreased in by 5.5% 2020 compared to 2019. The main reason behind this might be associated with lockdown situations and less person-to-person infection transmission. However, the antibiotic usage patterns changed, and the use of certain antibiotics has markedly increased in 2020 compared to 2019, such as azithromycin which increased by 74%, suggesting that the COVID-19 pandemic might lead to unnecessary antibiotic prescriptions. In addition, third-generation cephalosporin use was more prevalent in 2020 than in 2019 [36].

The SMA has been a global concern, as it can cause public health problems, such as antibiotic resistance and drug toxicity [8,37]. Therefore, the significance of the self-medication impact of COVID-19 in any region can have an influence on the prevalence of such problems. Generally, the SMA was reported in several studies around the world including in developed and developing countries [33,34,38,39,40].

Briefly, no definitive patterns of SMA resulting from the pandemic can be formulated from the data analyzed from similar studies in literature hitherto. However, by comparatively assessing studies before and after COVID-19, pharmacists are less likely to dispense antibiotics now than ever, owing to the symptoms associated with the disease. Moreover, increased dispensing practices of specific antibiotics have been reported on account of articles, published guidelines, and clinical shreds of evidence on the use of azithromycin, for example, for COVID-19 infections due to its potential “anti-viral mechanisms” [38,39].

### 4.1. Factors behind SMA in EMR

In Iran, when a sample of women was assessed for their attitudes toward self-medication, out of 360 participants, 41% judged the practice of self-medication as harmless. Additionally, age, area of residence, level of education, and health insurance status were significant factors for self-medication [28]. Moreover, a study in Egypt reported that 53.6% of the 160 participants believed the main motive behind self-medication was cost-saving, with the most frequent self-administered drugs being analgesics (59.5%) and antibiotics (23.5%) [40]. Additionally, the economic instability in the EMR has resulted in poor jurisdictions that have ultimately facilitated the habit of SMA [41].

Patterns of self-medication differ between various populations and regions and are affected by different factors [42]. Self-medication is a frequent practice in countries with less effective healthcare systems and improper laws and regulations with regards to medication. Constraints on human resources, long waiting times in healthcare facilities, shortage of essential medicine, lack of available beds and spaces in healthcare facilities, and frequent closure of healthcare facilities are among the factors that lead to a higher prevalence of self-medication in such countries. It can also be influenced by multiple factors, including availability of the drugs, ease of accessibility without time limits, and better convenience in accessing drugs compared to seeking a healthcare professional.

One of the groups of individuals associated with a higher prevalence of self-medication are qualified healthcare professionals as it gives them the freedom of treating minor ailments and managing their health problems independently. Since COVID-19 was declared a global concern, an abundance of healthcare facilities worked overtime under stressful conditions, causing an increase in stress and anxiety, leading to an overall psychological burden, ultimately triggering self-medication to cope with the resulting stress and pressure [43,44,45]. Based on a study in Pakistan during the pandemic’s peak, fear of being exposed to the coronavirus in hospitals and clinics increased to an extent that people preferred to avoid their routine clinic visits and consultancy and turn toward self-medication. This issue is specifically higher in people suffering from diseases such as diabetes, hypertension, allergy, digestive disorders, etc., compared to people with no diseases as they believe coronavirus exposure is a high risk to their condition [46].

The adverse effects of self-medication especially with antibiotics during the COVID-19 pandemic in EMR countries are still unexplored, warranting further assessment and reporting. This is needed because the consequence of this issue is an important public health problem and can lead to unfavorable complications.

Our study hints at the altered SMA practices upon the emergence of COVID-19. The prevalence of SMA reported in the EMR pre-COVID-19 ranged widely from 19 to 82% [6,7,18]. Our findings of SMA studies from EMR countries during COVID-19 report the prevalence of SMA practices spans from 20.8% to 45.8% [26,29]. Nonetheless, several factors led to the initiation of SMA [40,42,44,45,46]. Upon such bases, we suggest that SMA practices decreased during COVID-19, but the lack of sufficient studies reporting such in the EMR makes these findings inconclusive. However, patterns of increased azithromycin use were evident during COVID-19. Out of the nine studies reported, four of them assessed and reported the use of azithromycin in the context of self-medication, while pre-COVID, penicillin, cephalosporins, and fluoroquinolones were reported as being more prevalent [8].

In countries other than those that fall under the EMR, studies have been assessing self-medication practices, especially where such practices are foreseeable. In the West African country of Togo, for example, 955 participants were assessed for such practices. The prevalence of this outcome variable was 34.2%. Interestingly, only 1.2% of the participants reportedly used azithromycin [34]. This could be because the study was conducted during the first few months of the pandemic. In addition, the low prevalence of the use of azithromycin could be explained by its relatively high cost [34]. In another African country (Nigeria), SMA has been considered a factor behind increased death rates due to COVID-19 [47]. A study in Nigeria (*n* = 461), they reported fear of emergency illness, stigmatization, being quarantined, fear of contact with an infected person, and delaying hospital services were the main reasons for SMA. Amoxicillin and ciprofloxacin (24.9% and 14.6%, respectively) were the most used antibiotics for the prevention and treatment of COVID-19 infection [48]. In Peru, the southern American country, which was among the top five countries reporting a high number of COVID-19 death, a study (*n* = 3792) reported that more than 34% of the respondents self-medicated. An estimated 4.8% consumed azithromycin, and 2.3% penicillin [35].

### 4.2. Limitations

The lack of research and published studies on SMA in EMR affects the study’s generalizability, not only because of the paucity of real data and statistics analyzing antibiotic use in this region, but also because most of the included studies were cross-sectional studies such as those found in the searches. SMA analysis during COVID-19 was performed for different countries at different time points (during the two years of the COVID-19 pandemic 2020 and 2021) with fluctuations in the frequency of infection; a more robust conclusion would have been obtained if the SMA use data were compared in parallel for each country before and during the pandemic, which would have been possible had there been no dearth of literature.

## 5. Conclusions

The prevalence of self-treatment with antibiotics was high during the COVID-19 pandemic in many EMR countries. However, there was a decrease in the SMA compared to pre-COVID-19 pandemic with increased dispensing of specific antibiotics during the COVID-19 period. Many factors were reported, such as fear from infection, quarantine, and cost-saving. This emphasizes the need for initiatives to implement educational programs among the EMR population. Such educational initiatives are needed, given that most countries in the region fail to adhere to the prescription-only protocol of antibiotic dispensing and provided open access to antimicrobials on patients’ demands. In addition, more studies of self-medication patterns along with their outcomes in the context of a pandemic will help prevent a further surge in inappropriate use of antibiotics and, hence, adverse events.

## Figures and Tables

**Table 1:** Summary of studies (*n* = 6) assessed self-medication with antibiotics (SMA) during COVID-19 among general population.

Authors, Year	Aim of the Study	Country	Sample Size	Main Findings	Main Common Reasons of SMA
Al-Taie et al., 2021 [26]	Assessing the attitudes, knowledge, and prevalence of self-medication use of antibiotics during the COVID-19 pandemic in different districts within the province of Baghdad.	Iraq	384	Fewer than half of the participants (45.8%) reported SMA without a prescription and they did not feel the need to complete the antibiotic course after symptom alleviation. The most common non-prescription antibiotics were oral amoxicillin, azithromycin, and cephalexin.	Flu/common cold and sore throat represented the common medical conditions for antibiotics intake without prescription.
Azhar et al., 2021 [24]	Assessing the attitudes, knowledge, and prevalence of self-medication during the COVID-19 pandemic in the province of Punjab.	Pakistan	290	SMA was reported in 21.5% of the study participants. Azithromycin was the common drug used among the participants.	- Fear of getting into contact to the virus - Unavailability of doctors - Normally self-treatment (habit)
Elayeh et al., 2021 [27]	Evaluating patterns and factors that affect self-medication practices in the country during the COVID-19 pandemic.	Jordan	1179	About 30% of participants self-medicated with antibiotics during the pandemic. Azithromycin was the common drug used among the participants.	- To treat symptoms such as fever, muscle pain, and sore throat.
Heshmatifar et al., 2021 [28]	Investigating factors affecting the self-medication for COVID-19 prevention in the elderly.	Iran	360	More than a quarter (27.1%) of the participants self-treated with antibiotics to prevent COVID-19.	- Disease prevention - Home quarantine - Financial problems - Experiencing previous self-medication - Others’ advice
Heydargoy, 2020 [29]	Investigating the effect of COVID-19 on the use of antibiotics.	Iran	168	Less than a quarter (20.8%) of the participants self-medicated on antibiotics during the outbreak compared to the 38.1% who used antibiotics without a medical prescription before COVID-19.	- The outbreak of COVID-19 and quarantine - Fear of coronavirus disease
Yasmin et al., 2022 [25]	Determining and analyzing the prevalence of self-medication practices among medical students in Pakistan.	Pakistan	489	About 30% of participants self-medicated with antibiotics during the pandemic.	The reasons reported for self-usage of the medications included cold/flu, as preventive measures for COVID-19, and self-medication for symptoms.

**Table 2:** Summary of studies (*n* = 3) assessed self-medication with antibiotics (SMA) during COVID-19 in community pharmacies.

Author, Year	Aim of Study	Country	Sample Size	Main Findings
Abdelmalek and Mousa, 2022 [32]	Assessing misuse of azithromycin during the COVID-19 pandemic	Jordan	184	During COVID-19, pharmacists significantly dispensed more azithromycin (127%) without prescriptions than before the pandemic.
Elsayed et al., 2021 [30]	Describing antibiotic misuse and assessing its contributing factors to pharmacists’ infection preventive practices	Egypt	413	Less than a quarter of antibiotics (18%) was dispensed without prescriptions. However, 67% of the pharmacists stated that patients were more likely to be given antibiotics for showing any sign or symptom of COVID-19 infection.
Khojah, 2022 [31]	Investigating sales generated through dispensing non-prescription antibiotics and assessing pharmacists’ triaging skills for COVID-19 suspects	Saudi Arabia	120	About 16% of the study participants sold nonprescribed antibiotics owing to client demands and around 24% were not bothered by potential COVID-19 suspects.
